# Genome-wide identification and analysis of *Japonica* and *Indica* cultivar-preferred transcripts in rice using 983 Affymetrix array data

**DOI:** 10.1186/1939-8433-6-19

**Published:** 2013-08-10

**Authors:** Ki-Hong Jung, Hyun-Jung Gho, Hoi-Khoanh Giong, Anil Kumar Nalini Chandran, Quynh-Nga Nguyen, HeeBak Choi, Tian Zhang, Wen Wang, Jin-Hyun Kim, Hong-Kyu Choi, Gynheung An

**Affiliations:** Department of Plant Molecular Systems Biotechnology & Crop Biotech Institute, Kyung Hee University, Yongin, 446-701 Republic of Korea; CAS-Max Planck Junior Research Group on Evolutionary Genomics, State Key Laboratory of Genetic Resources and Evolution, Kunming Institute of Zoology, Chinese Academy of Sciences (CAS), Kunming, China; Department of Medical Bioscience, Dong-A University, Busan, Republic of Korea

**Keywords:** eQTL, *Indica*, *Japonica*, Gene ontolgy enrichment, Microarray meta-analysis

## Abstract

**Background:**

Accumulation of genome-wide transcriptome data provides new insight on a genomic scale which cannot be gained by analyses of individual data. The majority of rice (*O. sativa*) species are *japonica* and *indica* cultivars. Genome-wide identification of genes differentially expressed between *japonica* and *indica* cultivars will be very useful in understanding the domestication and evolution of rice species.

**Results:**

In this study, we analyzed 983 of the 1866 entries in the Affymetrix array data in the public database: 595 generated from *indica* and 388 from *japonica* rice cultivars. To discover differentially expressed genes in each cultivar, we performed significance analysis of microarrays for normalized data, and identified 490 genes preferentially expressed in *japonica* and 104 genes in *indica*. Gene Ontology analyses revealed that defense response-related genes are significantly enriched in both cultivars, indicating that *japonica* and *indica* might be under strong selection pressure for these traits during domestication. In addition, 36 (34.6%) of 104 genes preferentially expressed in *indica* and 256 (52.2%) of 490 genes preferentially expressed in *japonica* were annotated as genes of unknown function. Biotic stress overview in the MapMan toolkit revealed key elements of the signaling pathway for defense response in *japonica* or *indica* eQTLs.

**Conclusions:**

The percentage of screened genes preferentially expressed in *indica* was 4-fold higher (34.6%) and that in *japonica* was 5-fold (52.2%) higher than expected (11.1%), suggesting that genes of unknown function are responsible for the novel traits that distinguish *japonica* and *indica* cultivars. The identification of 10 functionally characterized genes expressed preferentially in either *japonica* or *indica* highlights the significance of our candidate genes during the domestication of rice species. Functional analysis of the roles of individual components of stress-mediated signaling pathways will shed light on potential molecular mechanisms to improve disease resistance in rice.

**Electronic supplementary material:**

The online version of this article (doi:10.1186/1939-8433-6-19) contains supplementary material, which is available to authorized users.

## Background

*Oryza sativa*, Asian cultivated rice, is grown all over the world (Khush 1997). *Japonica* and *indica* are representative subspecies of *O. sativa*. *Japonica* and *indica* rice evolved from different ancestors and diverged about 0.2 ~ 0.44 million years ago (Sang and Ge 2007; Wei et al. 2012). Genome-wide analysis to elucidate the differences between *japonica* and *indica* will be useful to explain the evolutionary events that led to their distinct features. During cultivation, these subspecies have developed unique morphologies and characteristic agronomic traits. Although several studies have tried to explain the differences between *japonica* and *indica* at a certain developmental stage or under experimental conditions, data from these studies are quite limited in their ability to explain general differences between *japonica* and *indica*. For example, transcriptome analysis of the light response in leaf tissues from 3 *japonica* varieties (Nipponbare, TP309, and Kitaake) and an *indica* variety (IR64) revealed that about 10% of light-responsive rice genes differed between subspecies (Jung et al. 2008b). Affymetrix microarrays were used to compare 93–11 (*indica*) and Nipponbare (*japonica*) seedlings and identify gene expression-level polymorphisms between them (Liu et al. (2010). RNA-seq data analyses recently revealed genome-wide transcriptome data for 93–11 (*indica*)/Nipponbare (*japonica*) or Gla4 (*indica*)/Nipponbare (*japonica*) seedlings (Lu et al. 2010). A significant amount of expression data is also available for the Agilent platform for both *indica* and *japonica*. The RiceXPro database in particular provides a large collection of expression data on japonica (Sato et al. 2013). This database provides diverse views for expression analysis of rice genes in terms of anatomy, development, diurnal regulation, hormone response and laser-captured root cell types (Sato et al. 2013).

Expression quantitative trait loci (eQTLs) regulate expression of mRNA or protein and originate from transcript-level polymorphisms of a single gene with a specific chromosomal location. Recently, tb1 and tag1, QTLs with key roles in maize domestication, have been cloned and analyzed (Tsiantis 2011; Kim et al. 2012). An exciting finding was that transcription factors are cloned in both QTLs. The tb1 QTL has variable promoter sequences and expression levels in maize and teosinte, while a single amino acid difference in tga1 explains the phenotypic variations in kernel traits. Thus, differential gene expression and genetic sequences have been selected during domestication. High-throughput array-based methods to measure mRNA abundance have enabled the identification of numerous eQTLs in plants, animals, and humans. Microarray analysis of 110 recombinant inbred lines (RILs) derived from a cross between Zhenshan 97 and Minghui 63 revealed 26,051 eQTLs in rice shoots 72 hours after germination (Wang et al. 2010). The study revealed correlations between QTLs for shoot dry weight and eQTLs, indicating possible candidate genes for the trait. More interestingly, chemotherapeutic drug susceptibility-associated SNPs are more likely to be eQTLs than a random set of SNPs in the genome, suggesting that eQTLs could also explain phenotypic differences between *japonica* and *indica*. Since the whole rice genome was sequenced, rice researchers have made efforts to characterize individual genes. The O verview of Functionally Characterized G enes in R ice O nline database (OGRO) (http://qtaro.abr.affrc.go.jp/ogro) provides information on 702 functionally-characterized genes (Yamamoto et al. 2012). This information will be very useful to evaluate the significance of candidate genes identified from high-throughput analysis for the discovery or enhancement of agronomic traits.

In this study, we collected 983 Affymetrix microarray data entries from the NCBI gene expression omnibus or ArrayExpress and normalized all slides by the same normalization method. The data were then sorted by cultivar—595 from *indica* and 388 from *japonica*. Using significant microarray analysis (SAM) in the TIGR multi experiment viewer (MEV) toolkit, we identified 104 genes with preferential expression in *indica* (*indica* eQTLs) and 490 genes preferentially expressed in *japonica* (*japonica* eQTLs). Here, we present the identification and analyses of these eQTLs.

## Results and discussion

### *Japonica* or *indica* eQTLs identified from rice Affymetrix microarray data

**Figure 1:**
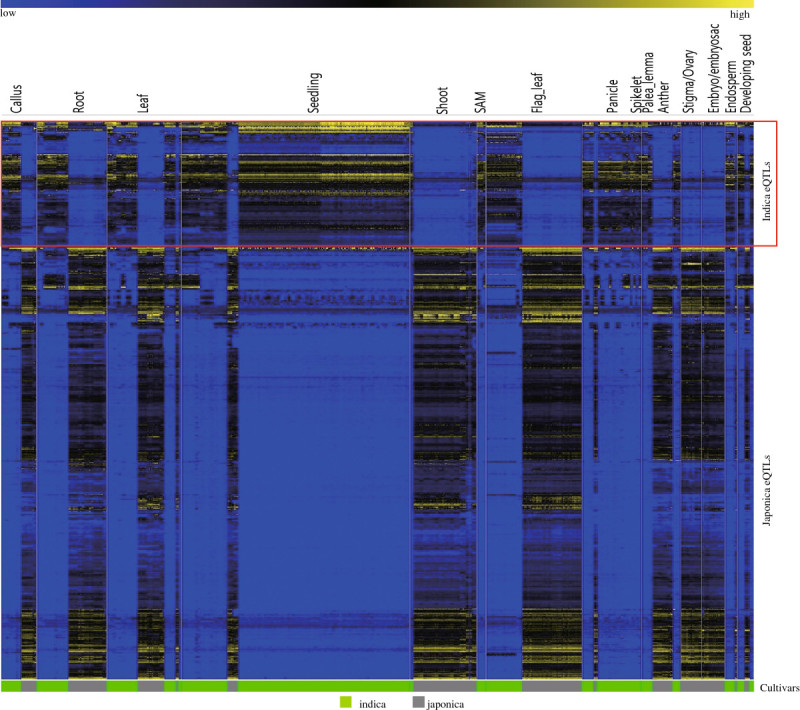
**Expression patterns of** ***japonica*** **and** ***indica*** **eQTLs identified using 983 affymetrix array data points.** From the rice Affymetrix microarray data available in the NCBI gene expression omnibus (GEO, http://www.ncbi.nlm.nih.gov/geo/), we used 983 arrays consisting of 595 *indica* and 388 *japonica* samples. Using a 2-fold change cut-off of *japonica* over *indica* expression and SAM analysis, we identified 490 loci showing preferential expression in *japonica* and 104 loci in *indica*. Blue color indicates low level of log_2_ intensity and yellow color indicates high level of log_2_ intensity. Green bar indicates *indica* samples and gray bar indicates *japonica* samples.

To identify eQTLs between *japonica* and *indica*, we collected Affymetrix microarray data from the NCBI gene expression omnibus (NCBI GEO, http://www.ncbi.nlm.nih.gov/geo/) or ArrayExpress (http://www.ebi.ac.uk/arrayexpress/) (Barrett et al. 2011; Parkinson et al. 2011). The total number of Affymetrix array entries was 983, as described in Additional file 1: Table S1; 388 entries were derived from *japonica*, and 595 were derived from *indica*. Array data associated with other species and those for which the subspecies were not identified were excluded. We normalized whole microarray data with the Affy package encoded using the R language (Gautier et al. 2004) and sorted by subspecies. We identified 4,685 probes with at least 2-fold differences in expression between *japonica* and *indica*. After performing significance analysis of microarrays (SAM) on the data installed in MultiExperiment Viewer (MEV) (Saeed et al. 2006), we identified 699 probes with preferential expression in *japonica* and 118 probes with preferential expression in *indica*. This resulted in 490 *japonica* eQTLs from 609 probes and 104 *indica* eQTLs from 118 probes. The number of eQTLs is less than that of corresponding probes because multiple probes target a single locus and some probes are unmapped to the chromosome. Therefore, we present expression profiles for the 490 *japonica* eQTLs and 104 *indica* eQTLs (Figure 1). The probes on the Affymetrix array platform are largely based on the Nipponbare genome sequence; thus, mRNAs from *japonica* might have higher affinity for the probes on this array platform. This could introduce bias in favor of *japonica* eQTLs. RNA-seq based on next-generation sequencing technology is expected to overcome the fixed-genome limitations of microarray technology. The expression patterns of *japonica* and *indica* samples were compared in 15 major categories of anatomical samples collected from 983 affymetrix arrays (Figure 1). Most candidate genes were differentially regulated between *japonica* and *indica* samples. Detailed information about the samples used in this figure is shown in Additional file 1: Table S1. In addition, we prepared the mapping data of 490 genes and 104 genes that are preferentially expressed in *japonica* and in *indica*, respectively, onto the 12 rice chromosomes (Additional file 2: Figure S1). Before carrying out further functional analysis, we confirmed the expression of 10 *japonica* and 7 *indica* eQTLs by reverse transcriptase (RT)-PCR (Additional file 3: Figure S2).

Recently, comparative analysis of genome expression in the heading-stage panicle from the six cultivated and wild rice lineages *Oryza sativa* subsp. *indica*, *japonica* and *javanica, O. nivara, O. rufipogon* and *O. glaberrima* was carried out. 5,116 genes differentially expressed in the heading-stage panicle of *japonica* and *indica* were identified (Peng et al. 2009). The large difference in the number of candidate genes identified in this analysis and in ours might come from differences in the range of analyzed samples and statistical criteria: we used 388 *japonica* and 595 *indica* samples, while Peng et al. (2009) used two biological samples prepared from the heading-stage panicle; we used SAM installed in MEV software, while Peng et al. (2009) used p-value > 0.7. Liu et al. (2010) compared 93–11 (*indica*) and Nipponbare (*japonica*) seedlings using methyl viologen (MV) as a reactive oxygen species agent in affymetrix microarrays. 232 probesets (213 genes) were identified with gene expression level polymorphisms between the two rice cultivars regardless of MV treatment through analysis using the supplemental data. Of these, we identified 55 probes (51 genes) showing more than 4-fold upregulation in *indica* compared to *japonica*, while 177 probes (162 genes) were more than 4-fold upregulated in *japonica* compared to *indica* (Additional file 4: Table S3). In this study, the number of genes preferentially expressed in *japonica* in the seedling stage is 3-fold more than the number of genes preferentially express in *indica*, identifying again the bias in favor of *japonica*-preferred genes. Of 162 genes preferentially expressed in *japonica* identified by Liu et al. (2010), 41 were also more than 4-fold upregulated in *japonica* samples when compared to *indica* samples from our analysis, while 5 of 51 genes preferentially expressed in *indica* had similar feature in our analysis (Additional file 4: Table S3). This data indicates that data on differential expression in *japonica* might be more stable than those in *indica*. In total, the differential expression patterns identified in the two cultivars in Liu et al. (2010) were much less than half repeated in our analysis, and the remaining significant genes might retain developmental stage-specific or stress-specific features. In summary, Peng et al. (2009) focused on analyzing genes differentially expressed in the heading-stage panicle between cultivated rice and wild rice, and Liu et al. (2010) provides genome-wide comparison between *japonica* and *indica* under stress (MV treatment) in the seedling stage. Compared to previous analyses, our analysis focused on identifying genes differentially expressed between *japonica* and *indica* through the whole life-cycle. Therefore, our data might be useful to determine general differences between *japonica* and *indica.* The differential expression patterns can be explained by deletion of *japonica* eQTLs in *indica* genome, suppression of *japonica* or *indica* eQTLs by defects in promoter or epigenetic regulation, and mismatches between *japonica* an *indica* sequences as indicated in Additional file 5: Table S2.

### Unknown genes and transposable elements are overrepresented in *japonica* and *indica* eQTLs

**Figure 2:**
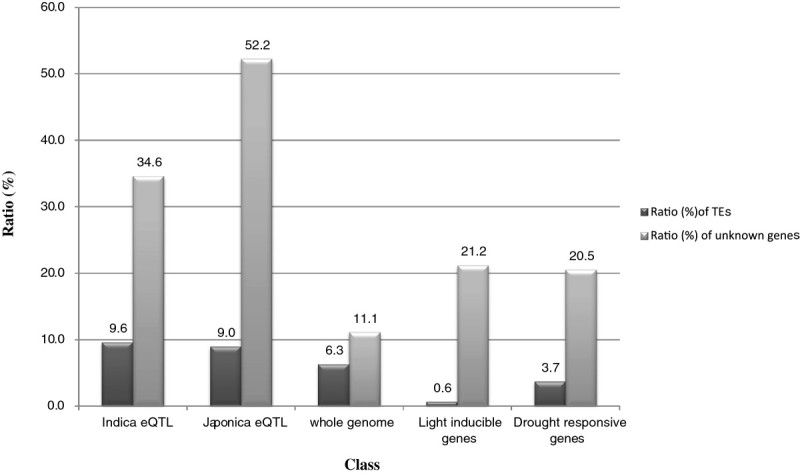
**Over-representation of unknown genes in** ***japonica*** **and** ***indica*** **eQTLs.** X axis indicates the gene classes with different features and Y axis indicates the relative ratio in each class. Dark gray bars indicate the relative ratios of TEs and weak gray bars indicate the relative ratios of unknown genes.

Of 490 *japonica* eQTLs, 181 entries are annotated with a gene function; the remainder are 256 unknown (expressed) genes, 9 hypothetical proteins, and 44 transposons/retro-transposons. Forty-nine of the *indica* eQTLs are annotated with a gene function and the others are 36 unknown genes, 9 hypothetical proteins, and 10 transposons/retro-transposons. Therefore, 36 (34.6%) *indica* eQTLs and 256 (52.2%) *japonica* eQTLs were unknown genes (Figure 2). Recently, the Rice Genome Annotation Project (RGAP) released annotations of 56,798 non-redundant loci, and 6311 (11.1%) of them are annotated as unknown genes (Figure 2). Therefore, we expect the *japonica* or *indica* eQTLs identified in this study to have novel functions. We also analyzed the relative ratio of transposable element-related genes (TEs, transposons, or retro transposons) and identified 10 (9.6%) in the *indica* eQTLs (104) and 44 (9.0%) in the *japonica* eQTLs (490). The ratios of TEs in *japonica* and *indica* eQTLs were much lower than those of the whole genome (15588/56798, 27.4%). However, due to redundancy among TEs, only 3579 were printed on the Affymetrix array (Jung et al. 2008a). If we multiply 3579/15588 and 27.4%, we find that only 6.3% of TEs are covered in the Affymetrix array. Therefore, TEs in both *indica* and *japonica* eQTLs were enriched about 1.5-fold more than expected; suggesting TEs might have significant evolutionary roles in traits that distinguish *japonica* from *indica*. Transcription of the TEs was generally weaker than that of their non-TE counterparts (Jiao and Deng 2007). We recently reported on light-responsive genes in a comparison of light- vs. dark-grown rice seedlings. Using criteria of at least a 2-fold upregulation in the light-grown seedlings and less than 10^-6^ false discovery rate p-value, we identified 7 TEs (0.1%) among 1108 light-responsive transcripts. This is a significantly smaller proportion than that found in *indica* and *japonica* eQTLs. Among 295 genes with at least a 4-fold higher drought-inducible expression (Plexdb, http://www.plexdb.org/), we identified 11 TE genes, a ratio (3.7%) about one-third of that found in *japonica* or *indica* eQTLs. These results indicate that the portion of active TEs under stress is significantly lower than that in *japonica* and *indica* eQTLs, suggesting the active role of TEs in the domestication of *japonica* and *indica*.

### Defense response is enriched in both *japonica* and *indica* eQTLs

**Figure 3:**
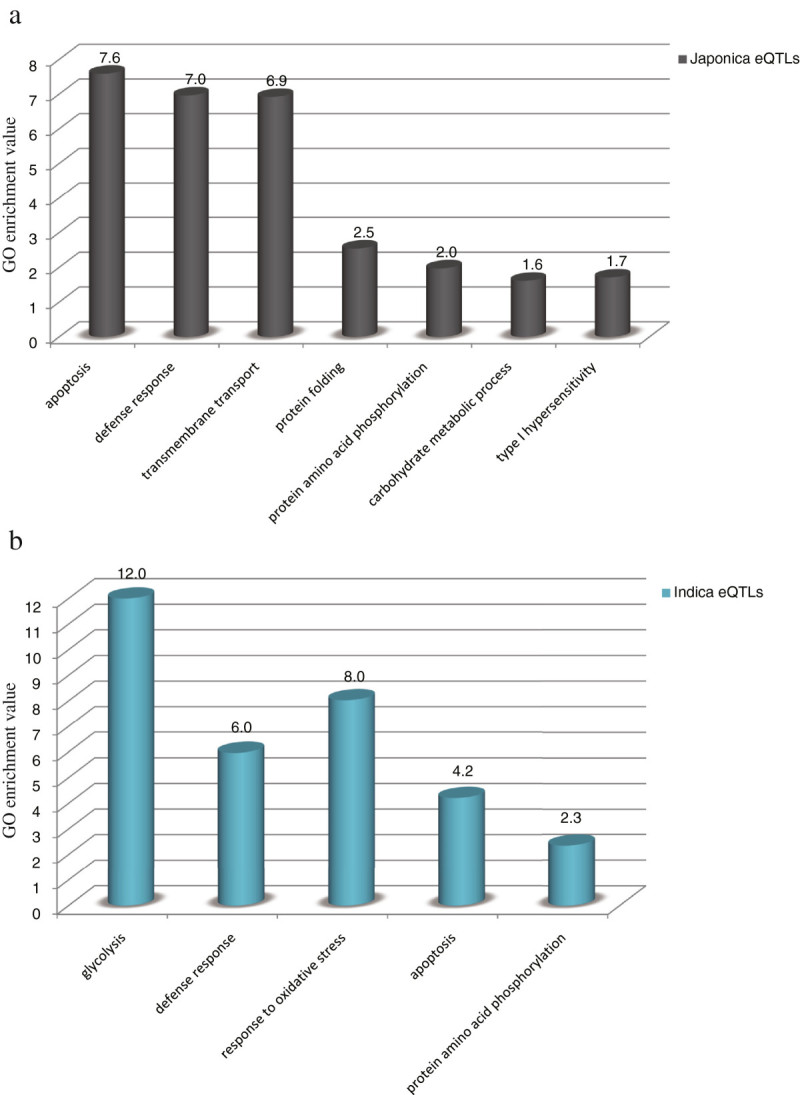
**Gene Ontology enrichment analysis of** ***japonica*** **and** ***indica*** **eQTLs.** The gene Ontology enrichment analysis tool installed in rice oligonucleotide array database (ROAD, http://www.ricearray.org/) was used to identify GO terms enriched in *japonica* **(a)** and *indica* **(b)** eQTLs. For each GO term, the GO enrichment value was the ratio of the number of observed genes in the gene list divided by the expected number, given the size in the gene list compared with the whole genome. X axis indicates the GO term and Y axis indicates GO enrichment value. The GO enrichment values were represented numerically in this figure.

GO enrichment analysis for *japonica* eQTLs identified at least a 2-fold greater enrichment of apoptosis, defense response, transmembrane transport, protein folding, protein amino acid phosphorylation, carbohydrate metabolism, and type I hypersensitivity genes (Figure 3a). GO enrichment analysis of *indica* eQTLs revealed that glycolysis, oxidative stress response, defense response, apoptosis, and protein amino acid phosphorylation genes are significantly enriched (Figure 3b). Genes involved in the defense response, apoptosis, and protein amino acid phosphorylation are significantly enriched in both *japonica* and *indica* eQTLs.

We found significant overlap between the defense response and apoptosis: 26 *japonica* eQTLs and 6 *indica* eQTLs were involved in these functions (Additional file 6: Table S4). More interestingly, 15 *japonica* eQTLs associated with the defense response are located on chromosome 11, suggesting that this chromosome may be a hotspot for enhanced disease resistance. Our findings correlate well with the finding that the sequences of rice chromosomes 11 and 12 are rich in disease resistance genes and recent gene duplications (Consortia. 2005). However, we cannot ignore the possibility that some of the selected genes can function negatively to disease resistance. Among the *indica* eQTLs associated with the defense response, we identified an NBS-LRR, 2 disease resistance protein RPM1s, a terpene synthase, a thionin family protein, and a transposon (Additional file 6: Table S4). NBS-LRR proteins are important for the resistance response to biotic challenges, especially for rice blast fungus (Jeung et al. 2007; Lin et al. 2008; Okuyama et al. 2011). Identification of NBS-LRR and RPM1 in both *japonica* and *indica* eQTLs indicates that both cultivars might have evolved unique NBS-LRR and RPM1 genes for their defense responses.

We identified 19 *japonica* eQTLs and 5 *indica* eQTLs associated with protein phosphorylation. Of the 19 *japonica* eQTLs, there are 11 receptor-like kinases, 2 wall-associated kinases, a DUF26 kinase, an O-methyltransferase, and an expressed protein. Of the *indica* eQTLs, there are 2 calcium-dependent protein kinases (CAMKs), 2 protein kinases, and a casein kinase. Receptor-like kinases and wall-associated kinases are characteristic of *japonica* eQTLs and CAMKs are unique to *indica* eQTLs.

Transcription regulation is generally affected by upstream phosphorylation cascades. We identified 4 *japonica* eQTLs associated with transcriptional regulation (i.e., *Os04g35010* encoding bHLH, *Os05g25770/OsWRKY45* and *Os11g45850/OsWRKY81* encoding WRKY TF, and *Os12g07950* encoding transcriptional regulator Sir2 family protein), and 3 *indica* eQTLs (i.e., *Os06g30090* encoding bHLH, *Os08g18000* encoding a F-box domain-containing protein, and *Os10g26940* encoding a BURP domain-containing protein) (Additional file 6: Table S4). OsWRKY45 confers enhanced resistance to fungal pathogens (Shimono et al. 2007). *OsWRKY45-1* (*japonica* eQTL) - overexpressing plants showed increased susceptibility, and *OsWRKY45-1* - knockout plants showed enhanced resistance to bacterial pathogens *Xanthomonas oryzae pv oryzae (* Xoo*)* and *Xanthomonas oryzae pv oryzicola (Xoc)*. *OsWRKY45-2* (*indica* eQTL)-overexpressing plants showed enhanced resistance and *OsWRKY45-2*-suppressed plants showed increased susceptibility to *Xoo* and *Xoc*. This suggests that *OsWRKY45-1* and *OsWRKY45-2* confer distinct disease resistance to bacterial pathogens (Tao et al. 2009).

Protein folding, transmembrane transport, carbohydrate metabolism, and type I hypersensitivity genes are exclusively enriched in *japonica* eQTLs (Additional file 6: Table S4). In contrast, glycolysis and the oxidative stress response are uniquely enriched in *indica* eQTLs. The smaller population of *indica* eQTLs identified in this study may explain the smaller number of GO terms enriched in *indica* eQTLs. Johns and Mao (2007) carried out gene ontology analysis for polymorphism levels between putative homologues in *japonica* and *indica*, identifying four functional classes: genes involved in production of defense-related compounds, cell wall synthesis, cell signaling, and transcription factors. At least 6% of the *japonica* and *indica* genomes are unusually divergent (Tang et al. 2006). Of GO terms associated with biological processes in highly-divergent regions of *japonica* and *indica*, response to biotic stimulus is the most significantly overrepresented (Tang et al. 2006). These results further highlight the significance of the defense response GO term enriched in both *japonica* eQTLs and *indica* eQTLs.

### Biotic stress overview in MapMan toolkit revealed key elements of the signaling pathway for the defense response in *japonica* and *indica* eQTLs

**Figure 4:**
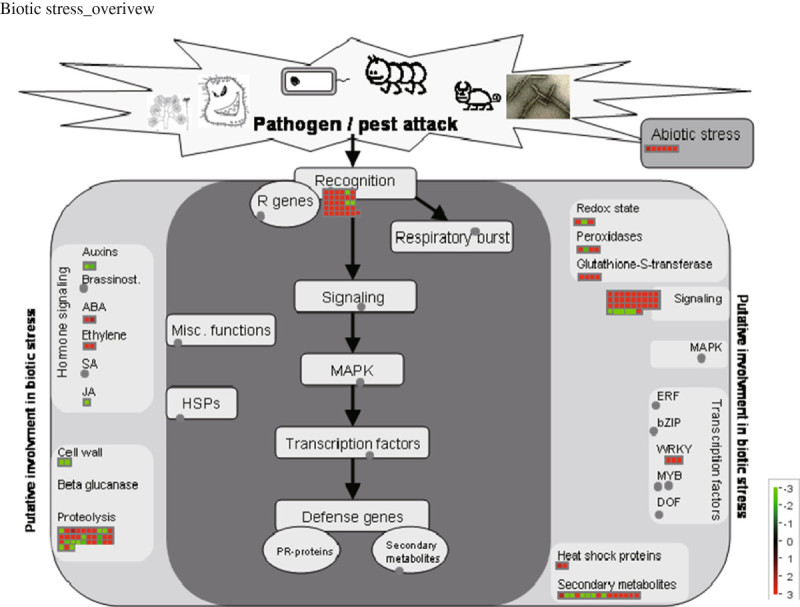
**MapMan analysis of** ***japonica*** **and** ***indica*** **eQTLs.** Biotic stress overview installed in the MapMan toolkit after integration of log_2_ fold change data of average *japonica* intensity over average *indica* intensity. Red color indicates upregulation in *japonica* cultivar and green color indicates upregulation in *indica* cultivar. Detailed information of *japonica* and *indica* eQTLs integrated in MapMan overviews is prepared in Additional file 7: Table S5.

MapMan is a useful tool for visualizing high-throughput omics data such as genome-wide microarray gene expression data or RNA-seq technologies in the context of metabolic pathways or cellular processes. Since GO enrichment analysis revealed that “defense response” genes were significantly overrepresented between both genes that were preferentially expressed in *japonica* and those in *indica*, we employed the biotic stress overview installed in the MapMan toolkit. To do this, we uploaded log_2_-fold change data of the average expression level of *japonica* samples over the average expression level of *indica* samples in Additional file 5: Table S2 to the cellular process overview and biotic stress overview installed in MapMan (Figure 4). Then, we mapped the fold-change data to MapMan using the Affymetrix probeset_ids in Additional file 5: Table S2. It is also possible to map rice genome annotation project (RGAP) locus ids in the MapMan tool. We mapped 594 genes on the Affymetrix array to MapMan terms (Additional file 7: Table S5).

Two hundred and forty-four genes have MapMan terms with assigned functional classifications but the others (350 genes) do not, which further demonstrates the high frequency of unknown genes in *japonica* and *indica* eQTLs (Figure 2). Figure 4 suggests detailed components of the signaling pathway in the biotic stress overview. The biotic stress overview feature begins with pathogen recognition by R genes, followed by respiratory burst, heat shock proteins, miscellaneous function, signaling, MAPK cascades, transcription factors, defense response by pathogen-related (PR) proteins, protein degradation, and secondary metabolites (Figure 4).

#### Pathogen recognition by R genes

MapMan analysis for *japonica* and *indica* eQTLs revealed 31 elements relating to pathogen recognition by R genes, and these elements include 26 NB-ARC/NBS-LRR proteins, 2 disease resistance RPP13-like proteins, 2 Yr10 stripe rust resistance proteins, OsWRKY125, etc. (Additional file 7: Table S5). Four (*Os09g09750, Os06g22460, Os06g4939* 0, and *Os11g35490*) of them are *indica* eQTLs, and the others are *japonica* eQTLs. *Pia* (*Os11g11810*) encodes a NBS-LRR disease resistance protein preferentially expressed in *indica* and *Pi25/Pid3* (*Os06g22460*) encodes a NB-ARC disease resistance protein preferentially expressed in *japonica* that are known to be involved in blast tolerance (Shang et al. 2009; Chen et al. 2011; Okuyama et al. 2011). These results suggest that rice cultivars might develop unique defense mechanisms by selecting unique NB-ARC/NBS-LRR proteins. We expect that all remaining preferentially-expressed *japonica* and *indica* genes encoding NB-ARC/NBS-LRR proteins might play key roles in enhanced defense responses against blast or other pathogens.

#### Hormone signaling

We identified 5 elements related to auxin (Os07g38890 and Os03g62060), jasmonate (Os02g12690), abscisic acid (ABA) (Os07g18162), and ethylene (Os09g27820) signaling (Additional file 7: Table S5). The three genes relating to auxin and jasmonate signaling are *indica* eQTLs, and those relating to ABA and ethylene signaling are *japonica* eQTLs. We identified 3 elements related to redox reactions and 7 elements related to miscellaneous functions, including glutathione S-transferase and peroxidase (Additional file 7: Table S5). Two of them are *indica* eQTLs, and the others are *japonica* eQTLs.

#### Signaling pathways

Signaling pathways in general are understood as events occurring after recognition by R genes, although there are receptor-like kinases such as Xa21 and Xa26 that play roles as defense genes (Song et al. 1995; Sun et al. 2004). We identified 32 elements, including 25 receptor-like kinases (RLKs), 5 non-receptor kinases, a G-protein, and a calcium-binding molecule (Additional file 7: Table S5). Receptor-like kinases might have roles in the recognition of pathogen infection, as well as in signal transduction. Among the non-receptor kinases, we identified 2 Ca^2+^/calmodulin-dependent protein kinases (CAMKs; Os02g41580 and Os08g34240), a case in kinase II subunit alpha-1 (Os03g55389), a serine/threonine-protein kinase Arabidopsis Fus3-complementating gene 2 (AFC2; Os12g27520), and a protein kinase (Os03g53410). Calcineurin B may be either directly or indirectly linked with CAMKs. The ras family domain protein associated-G protein might have roles in the upstream signaling of diverse non-receptor kinases.

#### Transcription factors

Transcription factors (TFs) have roles in signaling pathways downstream of kinases. We identified 3 WRKY genes (*OsWRKY45*, *OsWRKY81*, and *OsWRKY125*) showing preferred expression in *japonica*. The roles of *OsWRKY45* in the defense response against pathogens have already been discussed. Phylogenetic comparison of rice and Arabidopsis WRKY families revealed that *OsWRKY81* was clustered with *AtWRKY18* in WRKY subgroup IIa, which functions in the positive regulation of basal defense and systemic acquired resistance (SAR) operating downstream from NPR1 in Arabidopsis (Wang et al. 2006). *OsWRKY125* contains both a NB-ARC domain, which is important in the recognition of pathogen attack, and a WRKY domain, which is important in transcriptional regulation of defense genes (Pandey and Somssich 2009). Arabidopsis RRS1-R, a chimera of a TIR-NB-LRR protein and a WRKY-type transcription factor, conferred resistance against multiple pathogens by the cooperation with the nuclear R protein RPS4 (Slootweg et al. 2010). Functions of several R genes such as MLA, RPS4, and snc1 are activated after nuclear localization. These findings suggest multiple roles for the *OsWRKY125* gene, including recognition of pathogens, signal transduction, and stimulation of defense gene functions.

#### Protein/cell wall degradation, secondary metabolism, and PR proteins

We identified 29 elements related to protein degradation (proteolysis), 16 related to secondary metabolism, 2 related to cell wall degradation, and a PR protein (Additional file 7: Table S5). Protein degradation is mainly performed by 2 methods: one depending on proteases such as cysteine protease (5 elements), aspartate protease (1 element), subtilase (1 element) and 2 other elements, and the other depending on proteasome cooperation with Skp, Cullin, and the F-box-containing complex (SCF). The substrate specificity of the latter is determined by a distinct F-box protein (Spencer et al. 1999). Twenty F-box proteins identified in this study belong to the SCF E3 ligase complex. 14 of these are *japonica* QTLs, and the others are *indica* eQTLs. Each QTL plays a unique function associated with biological processes or cellular events in the respective cultivars. In addition, we identified an Skp subunit (Os12g40300) of the SCF E3 ligase complex, 1 subunit (Os12g14840) of the Ring E3 ligase complex, 1 subunit (Os08g13130) of the BTB/POZ E3 ligase complex, and 1 subunit (Os03g37950) of a proteasome (Additional file 7: Table S5). The detailed features of ubiquitin- and autophagy-dependent degradation are presented in Additional file 8: Figure S3 and Additional file 7: Table S5. Secondary metabolism also plays significant roles in the defense response (Degenhardt 2009). Five types of secondary metabolites were identified: terpenoid (7 elements), phenylpropanoid (6 elements), flavonoid (1 element), carotenoid (1 element), and another (1 element). Cell wall degradation is considered as a mechanism to protect plants from biotrophs (Sun et al. 2011); 2 elements in our analysis were found to be associated with cell wall degradation (Additional file 7: Table S5). *PR* genes are well-known marker genes of pathogenesis or defense responses, and we identified 1 such *PR* gene (*Os08g28670*). The elements identified in this section might indicate candidate genes involved in defense signaling pathways in rice.

### Identification of functionally-characterized genes expressed preferentially in *japonica* or *indica*

To evaluate the functional significance of genes expressed preferentially in *japonica* or *indica*, we queried the OGRO database with 594 genes to see if any of them were functionally characterized. We identified 10 genes (Table 1). A blast resistance gene, *Pid3*, was identified from a cross between a rice blast-resistant *indica* variety and a susceptible *japonica* variety (Shang et al. 2009). The allelic *Pid3* loci in most of the tested *japonica* varieties were identified as pseudogenes due to a nonsense mutation at nucleotide position 2,208 starting from the translation initiation site. In addition, the expression of this gene was significantly repressed in *japonica* plants, suggesting another reason for the non-functionality of this gene in *japonica*. It was recently reported that the resistance of *Pia* originated from *japonica* rice (Cho et al. 2009), explaining its preferred expression in *japonica*. *Tiller Angle Control 1* (*TAC1*) controls tiller angle for dense planting during rice cultivation and was identified from crosses between an *indica* rice, IR24, which displays a relatively spread-out plant architecture, and an introgressed line, IL55, derived from *japonica* rice Asominori, which displays a compact plant architecture with extremely erect tillers (Yu et al. 2007). Heading date 6 (Hd6) encoding the alpha subunit of casein kinase II is a quantitative trait locus involved in rice photoperiod sensitivity and was identified from a cross between the *japonica* variety, Nipponbare and the *indica* variety Kasalath (Takahashi et al. 2001). A pair of allelic genes, OsWRKY45-1 and OsWRKY45-2, with a 10-amino acid difference, play opposite roles in rice resistance against bacterial pathogens and preferential expression of *OsWRKY45* in *japonica* provides another example of functional diversity (Tao et al. 2009). *Pia, Pid3/Pi25, OsWRKY45*, *TAC1*, and *Hd6* are good examples for evaluating the significance of genes expressed preferentially in *japonica* or *indica* during domestication.

**Table 1:** **Summary of functionally characterized** ***japonica*** **or** ***indica*** **preferred genes from OGRO (** http://qtaro.abr.affrc.go.jp/ogro **)**

MSU_ ID^a^	Gene name	Major category	Detailed function	Method^b^	log2 ja/in^c^	Reference
LOC_Os01g18860	OsSAMS3	Morphological trait	Dwarf	Kd^d^	−4.46 (in^g^)	(Li et al. 2011)
LOC_Os01g18860	OsSAMS3	Physiological trait	Flowering	Kd	−4.46 (in)	(Li et al. 2011)
LOC_Os01g18860	OsSAMS3	Physiological trait	Germination dormancy	Kd	−4.46 (in)	(Li et al. 2011)
LOC_Os01g18860	OsSAMS3	Physiological trait	Sterility	Kd	−4.46 (in)	(Li et al. 2011)
LOC_Os09g35980	TAC1	Morphological trait	Culm leaf	Nv^e^	−2.90 (in)	(Yu et al. 2007)
LOC_Os01g71930	Osg1	Physiological trait	Sterility	Kd	3.96 (ja^h^)	(Wan et al. 2011)
LOC_Os02g12380	HDA710	Morphological trait	Culm leaf	Kd	4.89 (ja)	(Hu et al. 2009)
LOC_Os02g12380	HDA710	Morphological trait	Dwarf	Kd	4.89 (ja)	(Hu et al. 2009)
LOC_Os02g12380	HDA710	Morphological trait	Panicle flower	Kd	4.89 (ja)	(Hu et al. 2009)
LOC_Os02g43370	OsYSL2	Physiological trait	Eating quality	Kd/Ox^f^	−3.03 (in)	(Ishimaru et al. 2010)
LOC_Os02g43370	OsYSL2	Resistance/Tolerance	Other soil stress tolerance	Kd/Ox	−3.03 (in)	(Ishimaru et al. 2010)
LOC_Os03g55389	Hd6	Physiological trait	Flowering	Nv	−4.13 (in)	(Takahashi et al. 2001)
LOC_Os05g25770	OsWRKY45	Resistance/Tolerance	Bacterial blight resistance	Kd/Ox	3.07 (ja)	(Tao et al. 2009)
LOC_Os05g25770	OsWRKY45	Resistance/Tolerance	Bacterial blight resistance	Kd/Ox	3.07 (ja)	(Shimono et al. 2012)
LOC_Os05g25770	OsWRKY45	Resistance/Tolerance	Blast resistance	Kd/Ox	3.07 (ja)	(Tao et al. 2009)
LOC_Os05g25770	OsWRKY45	Resistance/Tolerance	Blast resistance	Kd/Ox	3.07 (ja)	(Shimono et al. 2012)
LOC_Os05g25770	OsWRKY45	Resistance/Tolerance	Blast resistance	Ox	3.07 (ja)	(Shimono et al. 2007)
LOC_Os05g25770	OsWRKY45	Resistance/Tolerance	Cold tolerance	Kd/Ox	3.07 (ja)	(Tao et al. 2011)
LOC_Os05g25770	OsWRKY45	Resistance/Tolerance	Drought tolerance	Kd/Ox	3.07 (ja)	(Tao et al. 2011)
LOC_Os05g25770	OsWRKY45	Resistance/Tolerance	Salinity tolerance	Kd/Ox	3.07 (ja)	(Tao et al. 2011)
LOC_Os05g25770	OsWRKY45	Resistance/Tolerance	Sheath blight resistance	Kd/Ox	3.07 (ja)	(Shimono et al. 2012)
LOC_Os06g22460	Pi25	Resistance/Tolerance	Blast resistance	Nv	−3.38 (in)	(Shang et al. 2009)
LOC_Os06g22460	Pid3	Resistance/Tolerance	Blast resistance	Nv	−3.38 (in)	(Chen et al. 2011)
LOC_Os07g45570	OsBLE2	Morphological trait	Culm leaf	Kd	3.62 (ja)	(Yang et al. 2003)
LOC_Os07g45570	OsBLE2	Morphological trait	Dwarf	Kd	3.62 (ja)	(Yang et al. 2003)
LOC_Os07g45570	Aldolase	Morphological trait	Root	Kd	3.62 (ja)	(Konishi et al. 2004)
LOC_Os11g11810	Pia	Resistance/Tolerance	Blast resistance	Nv	5.56 (ja)	(Okuyama et al. 2011)

^a^ indicates the locus id provided from Rice Genome Annotation Project team at Michigan State University.

^b^ indicates methods used for the functional analysis.

^c^ indicates log2 fold change (averaged japonica intensity/averaged indica intensity).

^d^ indicates knockdown analysis through transgenic approaches or T-DNA insertion.

^e^ indicates natural variation.

^f^ indicates overexpression analysis through transgenic approaches.

^g^ indicates indica eQTLs.

^h^ indicates japonica eQTLs.

Unlike the above five genes, the functions of the other genes were identified through transgenic loss of function studies. *Rice brassinolide-enhanced gene 2* (*OsBLE2*) encoding fructose-bisphosphate aldolase is involved in the *brassinolide*-regulated growth and development processes in rice (Yang et al. 2003). This gene has been reported to regulate root development (Konishi et al. 2004). *Histone deacetylase 710* (*HDA710*) in rice regulates vegetative growth (Hu et al. 2009). *Rice beta-1,3-glucanase gene 1* (*Osg1*) is required for callose degradation in pollen development (Wan et al. 2011). *Rice yellow stripe 1-like gene 2* (*OsYSL2*) is required for the long-distance transport of iron and manganese and also contributes to eating quality (Ishimaru et al. 2010). Knock-down of *rice S-adenosyl-l-methionine synthetase 1, 2,* and *3* (*OsSAMS1, 2,* and *3*) showed pleiotropic phenotypes, including dwarfism, reduced fertility, delayed germination, and late flowering (Li et al. 2011). However, elucidation of the unique function of *OsSAMS3* may require gain of function analysis or the correlation to natural variation. Although the functions of the five genes in this section were not evaluated based on agronomic traits, these genes may have the potential to play key roles in domestication process.

## Conclusion

Meta-analysis based on the collection of genome-wide transcriptome data is useful to identify valuable information that is otherwise difficult to obtain from individual transcriptome data. In this study, we identified a large number of genes preferentially expressed in *japonica* and *indica* by analyzing Affymetrix array data comprising 595 *indica* and 388 *japonica* samples. Various types of epigenetic regulation mediated by miRNA, siRNA, histone modification, and insertion patterns of transposons might result in the differential expression of the identified eQTLs in *japonica* and *indica* (Slotkin and Martienssen 2007; Berdasco et al. 2008; Mallory and Bouche 2008; Byun et al. 2012). The most striking feature of this collection of genes is the dominance of genes with unknown function and TEs. It is interesting that there is a possible association between agronomic traits distinguishing *japonica* and *indica* cultivars and these novel genes. Recently, Gramene developed a web-based QTL database. From this database, 8646 QTLs were identified in rice. These data might be useful in association studies of candidate genes identified from genome-wide analysis and related agronomic traits. Even if the resolution of most QTLs is very low, the linkage information can help to connect the phenotype of gene-indexed mutants to agronomic traits. Functional analysis using T-DNA insertional mutants for genes preferentially expressed in *japonica* or *indica* is currently underway and will provide new insights into the evolution and domestication of *japonica* and *indica* cultivars.

## Methods

### Identification of *japonica* and *indica* eQTLs using 983 Affymetrix microarrays

The Affymetrix raw data for 983 arrays made with *japonica* and *indica* samples were downloaded from NCBI GEO (platform Accession Number is GPL2025) (Additional file 1: Table S1). Additional file 1: Table S1 summarized the accession number, cultivar name, variety name, and tissue/organ type of 983 slides based on the information in NCBI GEO or Arrayexpress. We used MAS 5.0 provided by the affy R package for Affymetrix arrays to convert probe-level data to expression values and normalize the expression values. The data were log_2_ transformed. We classified our data into 2 subgroups, namely, *japonica* (388) and *indica* (595), based on the cultivar name of samples used for the microarray experiments. We generated average log_2_ fold change of *japonica* samples over *indica* samples. SAM analysis in the TIGR Multi Experiment Viewer (MeV; http://www.tm4.org/mev.html) between japonica and indicia cultivars revealed 699 probes showing preferential expression in *japonica* and 118 probes showing preferential expression in *indica*. We identified 490 *japonica* eQTLs from 609 probes and 104 *indica* eQTLs from 118 probes (Additional file 5: Table S2). Selected genes in a cultivar showed at least 2.7 fold higher expression than in the other (Additional file 5: Table S2).

### Heatmap analysis

We used MeV to generate Heatmap expression patterns of 594 genes in Additional file 5: Table S2.

### Gene ontology term enrichment analysis

We evaluated the enrichment of GO (Gene Ontology) terms for preferentially expressed genes in *japonica* and *indica* (eQTL) in the biological process category. We calculated fold-enrichment for each GO Slim term in a querying gene list and identified GO terms and related gene entries with more than 2-fold GO enrichment and a hyper geometric p-value of < 0.05 (Additional file 6: Table S4). For each term, fold-enrichment is the observed number of genes in the gene list divided by the expected number of genes, given the size of the gene list compared with the whole genome. Additional file 9: Table S6 contains data of GO Slim terms selected by >2-fold enrichment and a hyper geometric p-value of < 0.05.

### MapMan analysis

Thirty-six MapMan BINs are currently used for the Rice MapMan classification, and these BINs can be extended in a hierarchical manner into subBINs (Usadel et al. 2005; Urbanczyk-Wochniak et al. 2006) (Additional file 7: Table S5). To integrate significant gene expression data from our transcriptome analysis into the diverse MapMan tools, we generated a dataset including Affymetrix probe_ids and average fold-change data of *japonica* over *indica*. For Figure 4, we used Cellular_response_overview and Biotic_stress_overview, and for Additional file 8: Figure S3, we used Proteasome Detail and Autophagy installed in the MapMan toolkit. The detailed procedure is described in a recent study by our group (Jung et al. 2012).

### RNA extraction and RT-PCR analysis

To evaluate the expression patterns of eQTLs identified in this study, we carried out RT-PCR for 17 candidates (10 *japonica* and 7 *indica* eQTLs). We grew seeds of Nipponbare and Dongjin (*japonica* cultivars) and IR8 and IR64 (*indica* cultivars) for a week and harvested the leaves of the plants for RNA expression analysis. RT-PCR was carried out as in a previous study (Jung et al. 2006). The reaction included an initial 5-min denaturation at 94°C; followed by 27 cycles of 94°C for 45 minutes, 60°C for 45 minutes, and 72°C for 1 minute; and a final 10 - minute extension at 72°C. Next, 20 μL of the reaction mixture was separated on a 1.2% agarose gel. The primers used for RT-PCR analysis are listed in Additional file 10: Table S7 and Additional file 11: Table S8.

## Electronic supplementary material

Additional file 1: Table S1: Summary of 983 Affymetrix microarray data points used in this study. (XLSX 36 KB)

Additional file 2: Figure S1: Chromosomal distribution of *japonica* and *indica* eQTLs. (JPEG 99 KB)

Additional file 3: Figure S2: RT-PCR analysis of 17 eQTLs to validate microarray data. (JPEG 81 KB)

Additional file 4: Table S3: List of genes preferentially expressed in *japonica* and *indica* that were identified in Liu et al. (2010) and comparison with our *japonica* and *indica* eQTLs. (XLSX 75 KB)

Additional file 5: Table S2: Average log_2_ fold change data of eQTLs used in Figure 1. (XLSX 66 KB)

Additional file 6: Table S4: Detailed information of genes identified by GO enrichment analysis. (XLSX 14 KB)

Additional file 7: Table S5: Detailed information of *japonica* and *indica* eQTLs used for MapMan analysis. (XLSX 43 KB)

Additional file 8: Figure S3: Ubiquitin and autophagy-dependent degradation overview with integration of *japonica* and *indica* eQTLs. (JPEG 941 KB)

Additional file 9: Table S6: Data used for GO enrichment analysis in Figure 3. (XLSX 12 KB)

Additional file 10: Table S7: Information on primers used to validate expression patterns of 17 selected eQTLs. (XLSX 11 KB)

Additional file 11: Table S8: Blast search result of primers in Table S7 using BGI rice genome sequence. (XLSX 12 KB)

Below are the links to the authors’ original submitted files for images.

Authors’ original file for figure 1

Authors’ original file for figure 2

Authors’ original file for figure 3

Authors’ original file for figure 4

## Abbreviations

ABA: Abscisic acid
CAMKs: Calcium-dependent protein kinases
eQTLs: Expression quantitative trait loci
GEO: Gene expression omnibus
GO: Gene ontology
ids: Identifiers
MeV: Multi experiment viewer
PR: Pathogen-related
RLKs: Receptor-like kinases
RT-PCR: Reverse transcriptase polymerase chain reaction
RILs: Recombinant inbred lines
ROAD: Rice oligonucleotide array database
SAM: Significant microarray analysis
SAR: Systemic acquired resistance
SCF: F-box-containing complex
TEs: Transposable elements
TFs: Transcription factors
