# Supplementary figures and images for: Genome-wide identification and analysis of Japonica and Indica cultivar-preferred transcripts in rice using 983 Affymetrix array data
Source: Rice (N Y). 2013 Aug 10;6:19. doi: 10.1186/1939-8433-6-19 (PMC4883688; doi:10.1186/1939-8433-6-19)

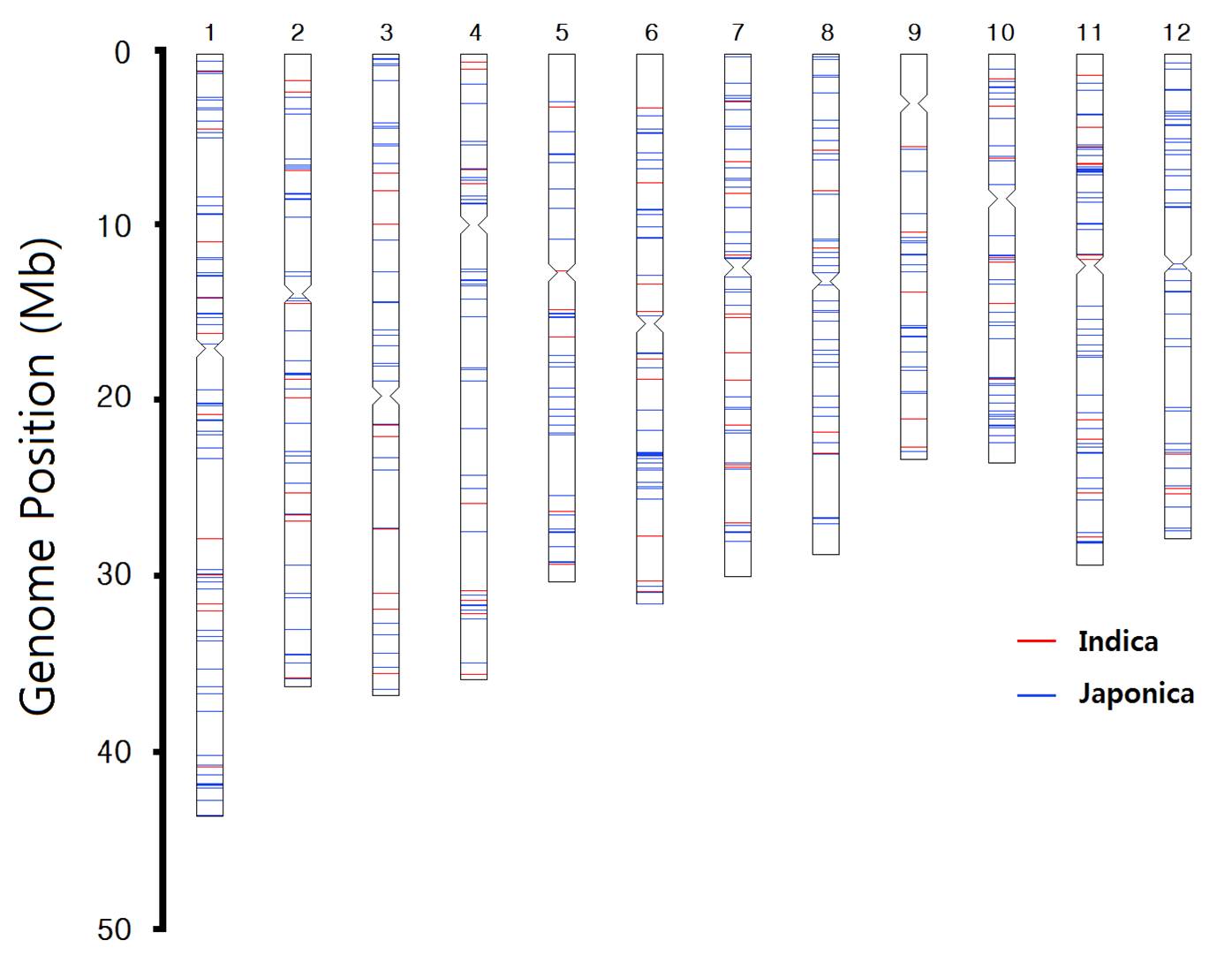

Supplement: Supplementary file 2 — Additional file 2: Figure S1: Chromosomal distribution of japonica and indica eQTLs. (JPEG 99 KB) [file 12284_2013_56_MOESM2_ESM.jpeg]

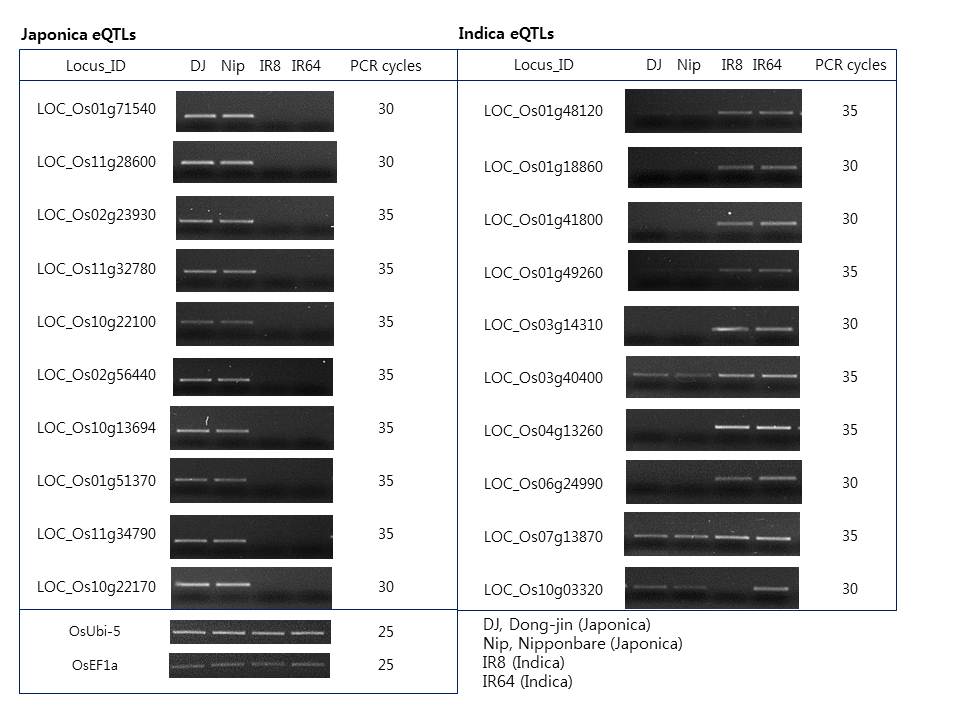

Supplement: Supplementary file 3 — Additional file 3: Figure S2: RT-PCR analysis of 17 eQTLs to validate microarray data. (JPEG 81 KB) [file 12284_2013_56_MOESM3_ESM.jpeg]

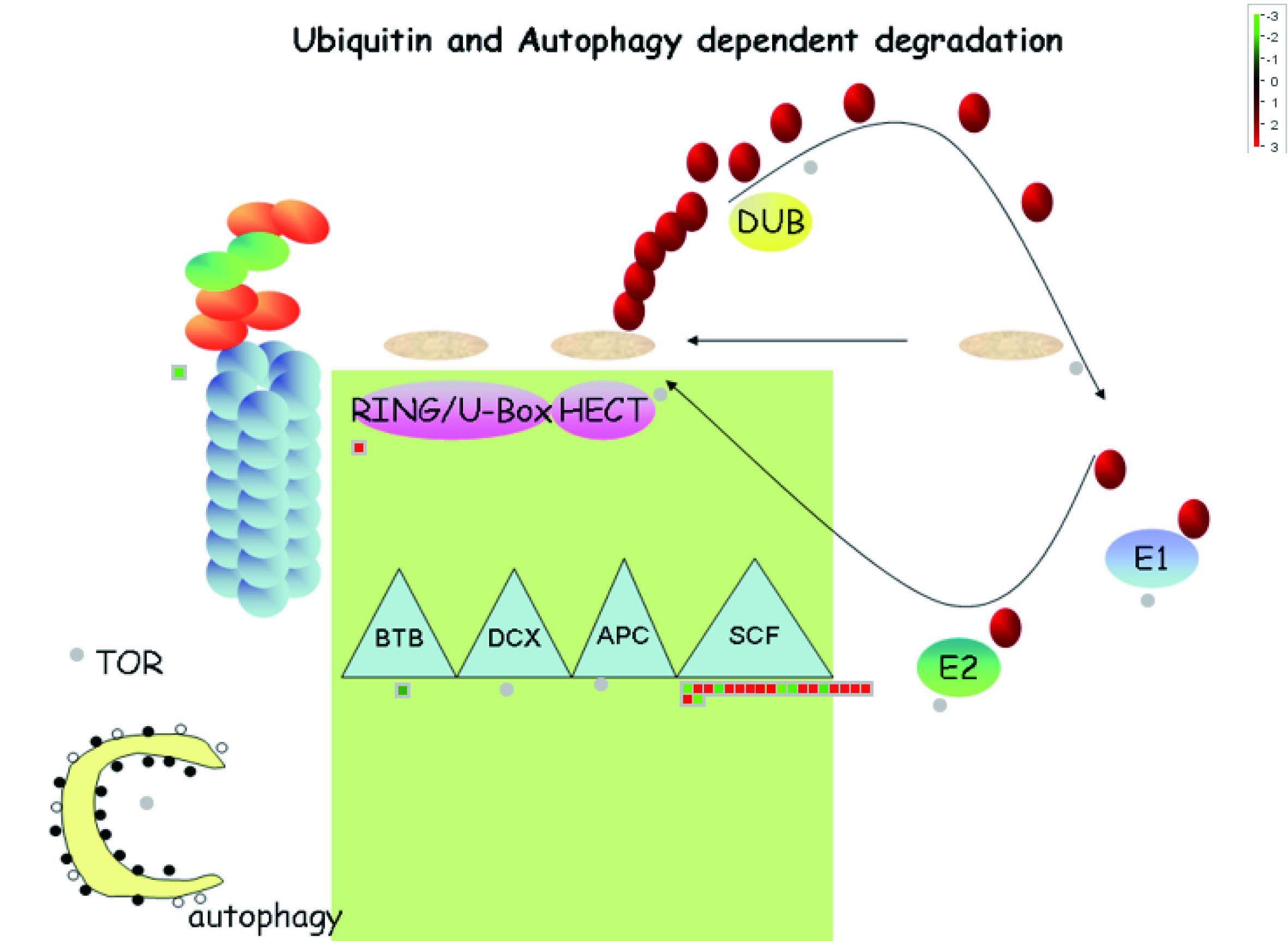

Supplement: Supplementary file 8 — Additional file 8: Figure S3: Ubiquitin and autophagy-dependent degradation overview with integration of japonica and indica eQTLs. (JPEG 941 KB) [file 12284_2013_56_MOESM8_ESM.jpeg]

low

high

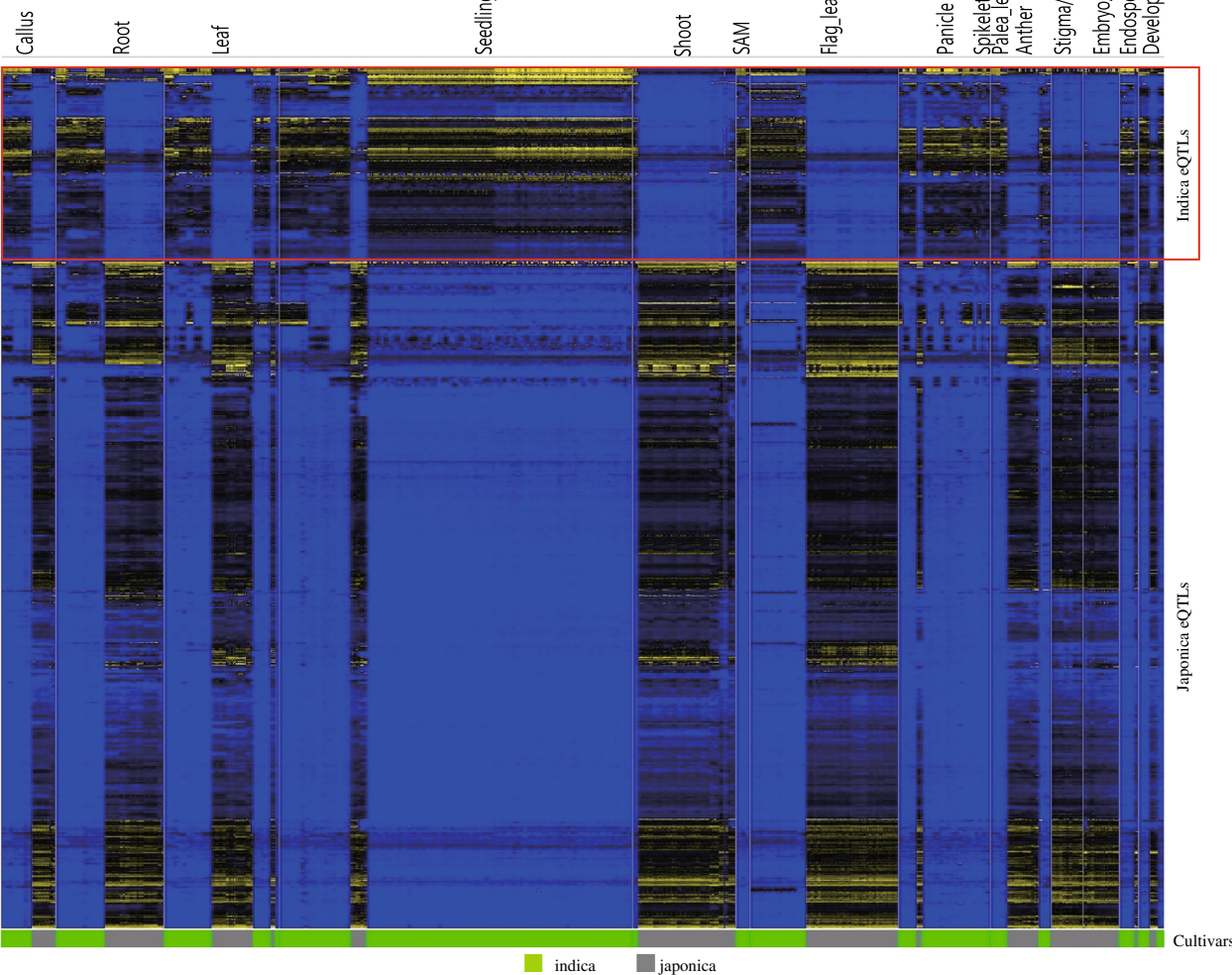

Supplement: Supplementary file 12 — Authors’ original file for figure 1 [file 12284_2013_56_MOESM12_ESM.pdf]

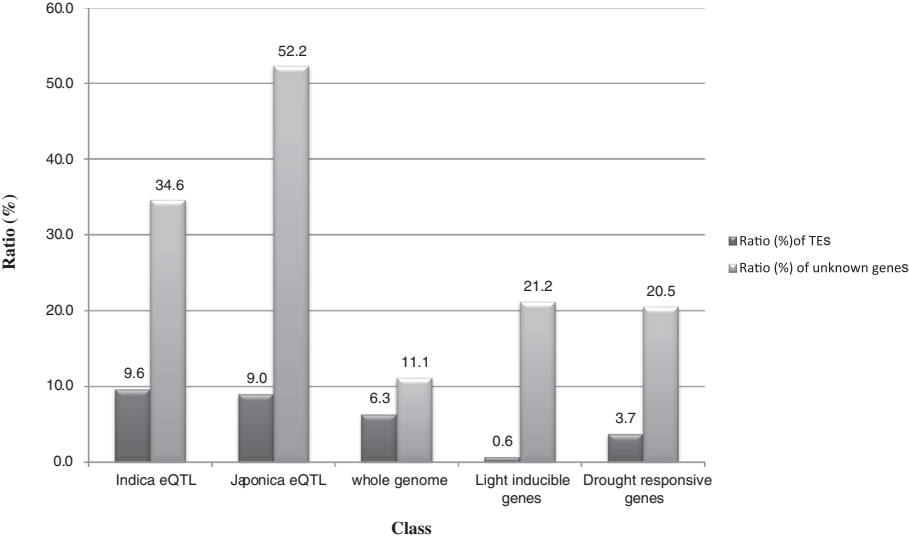

Supplement: Supplementary file 13 — Authors’ original file for figure 2 [file 12284_2013_56_MOESM13_ESM.pdf]

**a**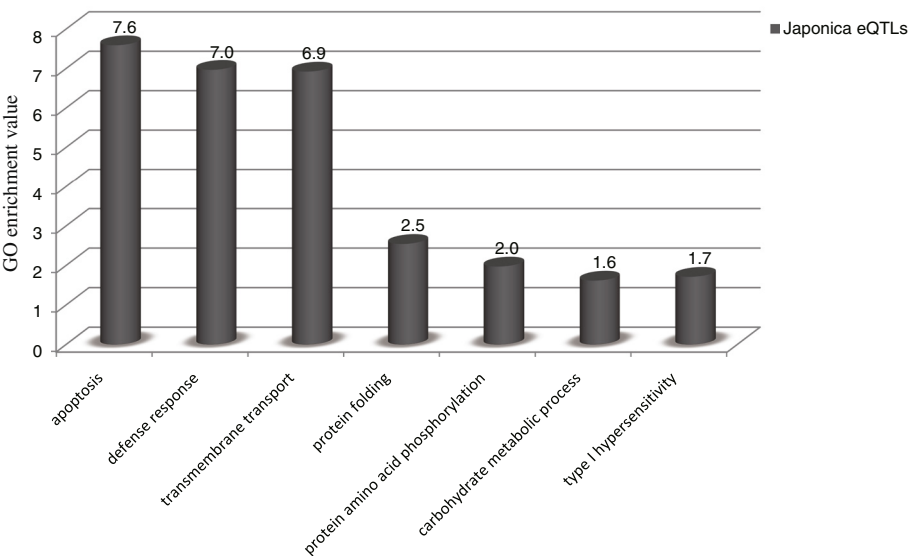**b**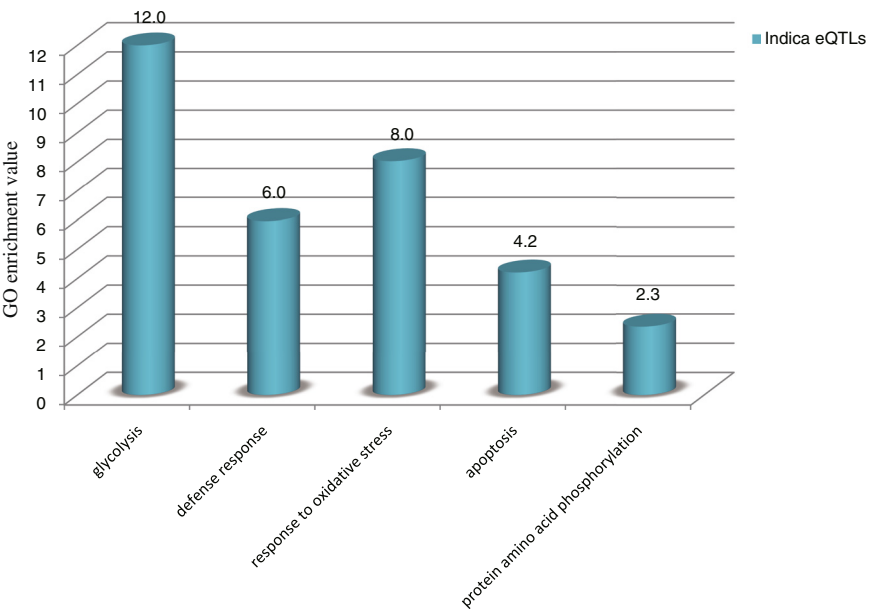

Supplement: Supplementary file 14 — Authors’ original file for figure 3 [file 12284_2013_56_MOESM14_ESM.pdf]

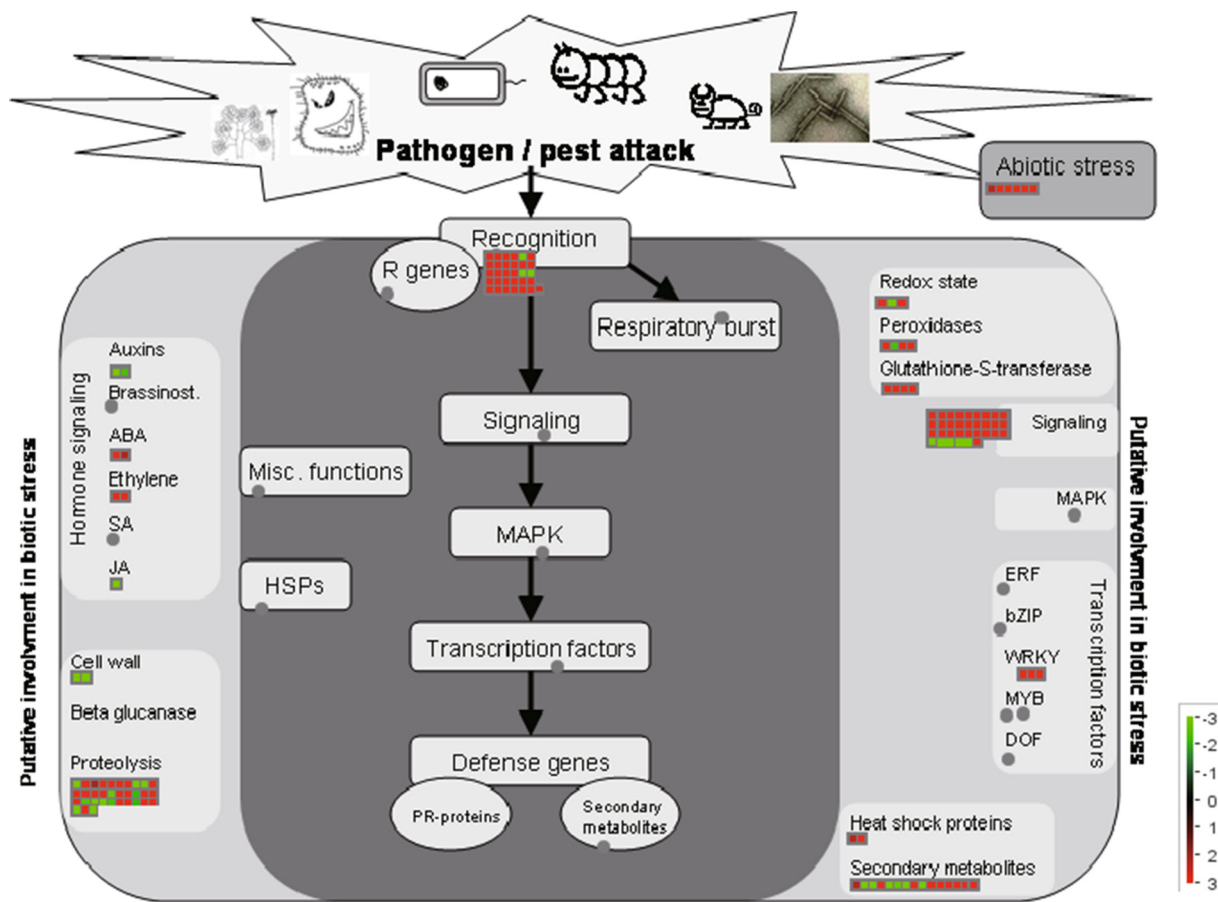

Supplement: Supplementary file 15 — Authors’ original file for figure 4 [file 12284_2013_56_MOESM15_ESM.pdf]
